# Simultaneously Optimize the Response Speed and Sensitivity of Low Dimension Conductive Polymers for Epidermal Temperature Sensing Applications

**DOI:** 10.3389/fchem.2020.00194

**Published:** 2020-03-19

**Authors:** Cheng Zhou, Ning Tang, Xiaoshuang Zhang, Ye Fang, Yang Jiang, Hainan Zhang, Xuexin Duan

**Affiliations:** State Key Laboratory of Precision Measuring Technology & Instruments, Tianjin University, Tianjin, China

**Keywords:** temperature sensor, nanowires, fast response, healthcare, tumor, smartphone

## Abstract

Low dimension poly(3,4-ethylenedioxythiophene) poly (styrenesulfonate) (PEDOT: PSS) has been applied as resistor-type devices for temperature sensing applications. However, their response speed and thermal sensitivity is still not good enough for practical application. In this work, we proposed a new strategy to improve the thermal sensing performance of PEDOT: PSS by combined micro/nano confinement and materials doping. The dimension effect is carefully studied by fabricating different sized micro/nanowires through a low-cost printing approach. It was found that response speed can be regulated by adjusting the surface/volume (S/V) ratio of PEDOT: PSS. The fastest response (<3.5 s) was achieved by using nanowires with a maximum S/V ratio. Besides, by doping PEDOT: PSS nanowires with Graphene oxide (GO), its thermo-sensitivity can be maximized at specific doping ratio. The optimized nanowires-based temperature sensor was further integrated as a flexible epidermal electronic system (FEES) by connecting with wireless communication components. Benefited by its flexibility, fast and sensitive response, the FEES was demonstrated as a facile tool for different mobile healthcare applications.

## Introduction

Real-time and continuous measurement of human body local epidermal temperature enables a better tracking of personal health status such as local wounds infection (Celeste et al., 2013), subcutaneous tumor (Sudharsan et al., 1999), as well as monitoring of body activities, since many diseases and physiological behaviors will cause local changes in body epidermal temperature (Deng and Liu, 2004; Helmy and Rizkalla, 2008; Ng, 2009; Li et al., 2017). Recently, wearable epidermal electronic systems (EESs) based on flexible devices have opened new frontiers in the measurement of body local temperature (Gao et al., 2014; Takei et al., 2015). Due to their soft and flexible nature, they can be directly attached to the human skin and conform to the body, local temperature can be detected anytime and anywhere (Webb et al., 2013; Zhang et al., 2016). Besides, the use of soft substrate enables high mechanical durability in different bending conditions, thus their responses will not be influenced by the movement of the body (Wu and Haick, 2018; Chang et al., 2019). However, due to the materials limitation, current EESs based thermal meters still suffer from the issues of responding slowly or not sensitive enough to body temperature change, which in fact cannot provide real-time precise temperature tracking. Hence the development of fast and sensitive response wearable temperature sensors which can track personal health status is still required.

Among all EES temperature sensor, PEDOT: PSS based resistor-type electronics have been largely applied since the PEDOT: PSS itself is very sensitive to temperature changes (Culebras et al., 2016) and the soft nature of such organic electronics ensures its excellent performance as flexible sensors (Lipomi et al., 2012). The thermosensitive mechanism of PEDOT: PSS can be explained by the structural change of PEDOT: PSS induced by the temperature change which eventually alters the conductivity of PEDOT: PSS (Takano et al., 2012; Zhou et al., 2014; Vuorinen et al., 2016). Previous studies on PEDOT: PSS based temperature sensors were focused on doping the PEDOT: PSS with other materials to enhance the device's response to temperature (Honda et al., 2014; Oh et al., 2018). However, understanding the control parameters regarding their response time to temperature change is less covered.

**Scheme 1:**
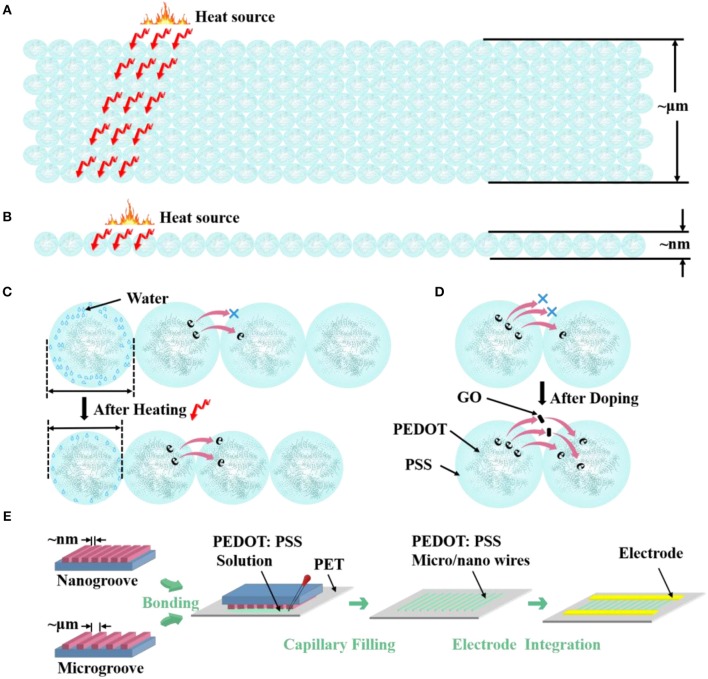
Morphological model shows the different S/V ratio of PEDOT: PSS **(A)** microwires and **(B)** nanowire. Morphological model of the **(C)** thermosensitive and **(D)** doping mechanism of PEDOT: PSS. **(E)** Carton shows the fabrication process of micro/nanowires.

In this work, in order to simultaneously optimize the response speed and sensitivity of conductive polymers for epidermal temperature sensing applications such as quick temperature detecting or real-time precise temperature tracking. we proposed a strategy to combine the micro/nano confinement with the materials doping to simultaneously optimize the response time and sensitivity of PEDOT: PSS based resistor-type temperature sensor. Low dimension PEDOT: PSS wires from a few micrometers down to sub-100 nm in diameters were fabricated using a low-cost micro/nanoscale printing approach (Gates et al., 2005; Massi et al., 2006; Duan et al., 2010; Tang et al., 2019). Their sensitivity and response time to temperature were compared (Scheme 1). Then, graphene oxide (GO) was selected to dope the PEDOT: PSS and the doping ratios were carefully optimized to enhance their temperature sensing performance. Hence the fabricated sensor under such strategy is responding both fast and sensitive enough to detective minute temperature change and track temperature in real-time. The sensor shows ultra-fast response and sensitive to body temperature change. Furthermore, the sensor is also used to achieve temperature monitoring of subcutaneous tumors in mice and by detecting the minute change of body temperature in mice to test the effect of drugs. Given the prominent mechanical and sensing properties, a homemade wearable system based on the temperature sensor was further developed to achieve a live and wireless transmission of the signals to a smartphone using Bluetooth assisted communication. These results demonstrate that this fast response skin-attachable nanowires-based temperature sensor has great potential as a wearable bioelectronic for application in medical diagnosis and mobile healthcare.

## Experimental Methods

### Reagents and Materials

mr-I T85 was purchased from Micro-Resist. PEDOT: PSS aqueous suspension (~1.3 wt%) with a conductivity of 1 S cm^−1^ was purchased from Sigma-Aldrich. GO was purchased from Chengdu Organic Chemical company and further sonicated for 30 min to form a uniform and stable dispersion with a concentration of 7 mg/ml. The compound solution was obtained by stirring PEDOT: PSS aqueous suspension and GO aqueous suspension for 10 min. Polydimethylsiloxane (PDMS) was purchased from Dow corning.

### Fabrication of Flat PDMS Molds With Micro/Nano Grooves

First, casting the liquid prepolymer of PDMS base and the curing agent in a 10:1 (w/w) ratio onto a silicon wafer with two kinds of silicon micro-ridges (3 μm in width and 1 μm in height and spacing 5 μm, 5 μm in width and 1 μm in height and spacing 10 μm, respectively). After the bubbles generated during the stirring process disappear, the wafer with the uncured PDMS was put into hot oven. After curing at 80°C for 40 min, the PDMS with microgrooves was cooled to room temperature and peeled off from the silicon wafer. Then, the PDMS with microgrooves was cut into pieces with the boundary of the microgrooves. The fabrication of the molds with nanogroove can be seen on previous case articles performed in our lab (Tang et al., 2019).

### Formation of Micro/Nano Channels

The molds with micro/nano groove were first treated with oxygen plasma (10 mTorr;10 sccm O_2_; 10 W; 15 s) to facilitate the contact between the molds and PET substrates. The molds were then bonded to the PET substrates which were also treated with oxygen plasma (20 mTorr;20 sccm O_2_; 20 W; 15 s). The molds can conformal contact with the PET substrates because of the oxygen plasma treated procedure and soft properties of the molds and PET substrates. Hence the micro/nano grooves changed into micro/nano channels after contacting.

### Electrodes Fabrication

The Au electrodes were then prepared by thermal evaporation on the PET substrate with nanowires under self-made copper shadow masks to promote the electrical connection. The electrodes were with pad length in 7 mm, width in 300 μm and spacing in 300 μm. The electrodes of microwires-based devices were silver paste at an interval of 3 mm approximately. Silver paste and silver wires were used to have further electrical contacts.

### Measurements

Atomic force microscope (AFM), Scanning electron microscope (SEM) and the confocal laser scanning microscope (CLSM) images were carried out with Dimension Icon (Bruker), FEI FP 2031/12 Inspect F50 and OLYMPUS OLS5000, respectively. Electrical measurements with a constant voltage of +1 V were performed with a semiconductor analyzer (Agilent Technologies, B1500A).

### Animal Tumor Model

Liver cancer tumor cells was injected into KM mice to establish an animal tumor model.

### Temperature Monitoring Based on Smartphone

The homemade wearable watch-type system was fabricated by integrating the AD5933/STM32/HC-05 and their peripheral circuits on a flexible circuit board. AD5933/STM32/HC-05 control resistance measurement, communication, and data transmission, respectively. An Android application program was developed on the smartphone to receive real-time data from the wearable watch-type system and plot the responses on the screen. Comparing the response change to its resistance calibration at standard temperature, the temperature was successfully measured.

## Results and Discussion

### Micro/nanowires Fabrication and Characterization

To study the structure effects on the thermal sensing performance of PEDOT: PSS, here we design three different sized PEDOT: PSS wires, where their widths vary from 4 μm to 70 nm (Scheme 1). Hence different S/V ratios are achieved. The micro/nanowires were fabricated by an adapted soft lithography which does not require any cleanroom facilities (Gates et al., 2005; Massi et al., 2006; Duan et al., 2010; Tang et al., 2019). Scheme 1E shows the schematic of fabricating procedure of micro/nanowires. After the micro/nanochannels (The preparation process is shown in the Experimental Methods) have completely formed, the PEDOT: PSS aqueous solution were dropped to the margins of channels. The solution filled the channels spontaneously by traction of the Laplace pressure. After the solvent has completely evaporated, removed the mold from PET and leaved the micro/nanowires on the PET substrate. Then, metal electrodes were fabricated on the side of the micro/nanowires through evaporation as the contact electrodes, thus resistor-type sensors have been achieved. The comparison between our method and current available methods for fabricating nanowires in the format of a table is shown in Supplementary Information.

**Figure 1:**
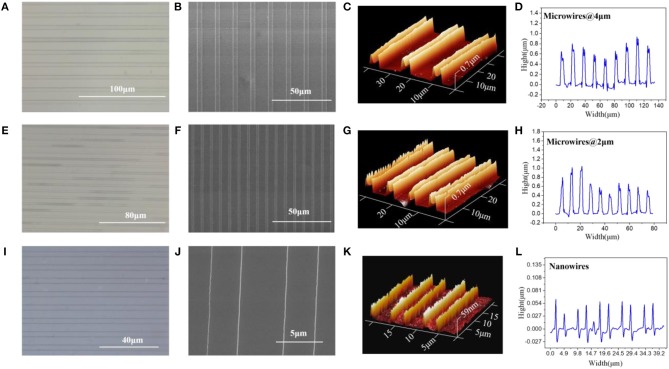
**(A)** Optical microscope, **(B)** SEM, **(C)** AFM, and **(D)** CLSM, image of microwires @ 4 μm. **(E)** Optical microscope, **(F)** SEM, **(G)** AFM, and **(H)** CLSM, image of microwires @ 2 μm. **(I)** Optical microscope, **(J)** SEM, **(K)** AFM, and **(L)** CLSM, image of nanowires.

Figure 1 shows the optical microscope, Scanning Electron Microscope (SEM), Atomic Force Microscope (AFM) and Confocal Laser Scanning Microscope (CLSM) images of the micro/nanowires with different sizes, respectively. These results show that the microwires have the same height (~ 0.7 μm) but with different width (4 μm, 2 μm). The nanowires have an average width of 70 nm and an average height of 59 nm. Thus, the S/V ratio of microwires @ 4 μm, microwires @ 2 μm and nanowires can be calculated as follow: 1.928, 2.429, and 45.52. As can be seen in the AFM images, the height of the microwires sides are higher than the middle part, which is due to the microchannels are treated with O_2_ plasma hence are more hydrophilic, thus the aqueous solution tends to stick to the wall before evaporates, which resulting in this phenomenon.

### Temperature Sensing

**Figure 2:**
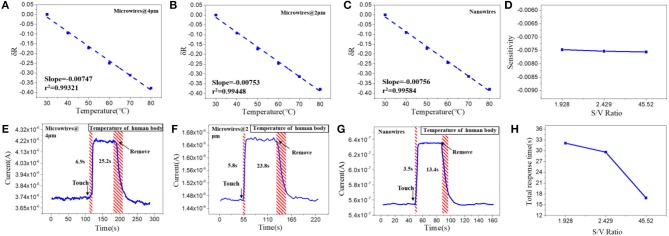
Relative resistance changes depending on the temperature with increments of 10°C of the **(A)** microwires @ 4 μm, **(B)** microwires @ 2 μm and **(C)** nanowires, based temperature sensor. **(D)** Sensitivity of the sensors with different S/V ratio. Real-time response of **(E)** microwires @ 4 μm, **(F)** microwires @ 2 μm and **(G)** nanowires, based temperature sensor. **(H)** Total response time of the sensors with different S/V ratio.

To study the structure effects on their temperature sensing behavior, we compared the response value and response speed of the three PEDOT: PSS based resistor-type temperature sensors with different S/V ratio. Their resistance were measured under a temperature range from 30 to 80°C with an interval of 10°C (Figures 2A–C). The response value is defined as:

$$\begin{array}{l} \;\delta \text{R}=\left(\left(\text{R}-{\text{R}}_{0}\right)/{\text{R}}_{0}\right) \end{array}$$

where R_0_ and R are the resistance values at 30°C and the set temperature. And TCR is defined as TCR = ((R−R_0_)/R_0_)/ΔT = δR/ΔT. The results show that the resistance of the three devices decrease linearly as the temperature rises and their sensitivity (slope of the liner fitting) are rather similar (Figure 2D). The δR of PEDOT: PSS microwires @ 4 μm, microwires @ 2 μm and nanowires-based temperature sensor between 30 and 80°C (303 and 353 K) are −0.377, −0.378, and −0.379, so the TCR of them can be calculated as −0.007545, −0.007579, and −0.007599 K^−1^. We then tested their response speed to temperature change by attaching or removing these devices to human arm which has a constant temperature. As shown in Figures 2E–G, the currents show increase or decrease after touching or removing the devices from the human skin. Compare with their thermal sensitivity, very differently, the devices with different S/V ratio show quite different temperature response speed (defined as the time required to reach 90% of the zenith response). The response and recovery time of the microwires @ 4 μm, microwires @ 2 μm and nanowires-based temperature sensor were 6.9 and 25.2 s, 5.8 and 23.8 s, 3.5 and 13.4 s, respectively. As compared in Figure 2H, a clear trend can be observed that the response speed increases with the increase of the S/V ratio, especially the nanowires with a maximum S/V ratio shows an ultra-fast response speed. This behavior can be explained by the electron transportation and thermal sensing mechanism of PEDOT: PSS which is very related to its microstructure. As shown in Scheme 1C, PEDOT: PSS has a typical core-shell structure in which the core is PEDOT nanocrystal and surrounded by PSS-rich shell. The insulating PSS boundaries with a strong hygroscopic ability have a major effect to the overall conductance of the PEDOT: PSS (Takano et al., 2012; Zhou et al., 2014). When temperature rising, the water molecules absorbed in the PSS boundaries will be partially released, which leads to the shrinkage of PSS boundaries and results in a decrease of the distance between adjacent PEDOT (Scheme 1C). This will facilitate the electron transportation between the PEDOT domains and results the decrease of the resistance of PEDOT: PSS (Zhou et al., 2014; Vuorinen et al., 2016). The response of PEDOT: PSS film are shown in Figure S1 in Supplementary Information. By confining the PEDOT: PSS from 2D thin film to 1D nanowire (increase of its S/V ratio), PSS boundaries can be fully exposed to the external environment, thus increase its thermal conduction and facilitates the water evaporation, which will increase its response speed to temperature change. Besides, the electron transport efficiency within the 1D nanowire is improved as well. Other nanomaterials-based resistive temperature sensors also have showed such advantages of nanomaterials in temperature detecting (Joh et al., 2018; Sehrawat et al., 2018; Bang et al., 2019; Cui et al., 2019). However, reducing the materials dimension will not influence the deformation ratio of the PSS boundaries, which is related to the amount of the water absorbed, thus the change of S/V ratio will not influence the thermal sensitivity of PEDOT: PSS.

### Doping Effect

**Figure 3:**
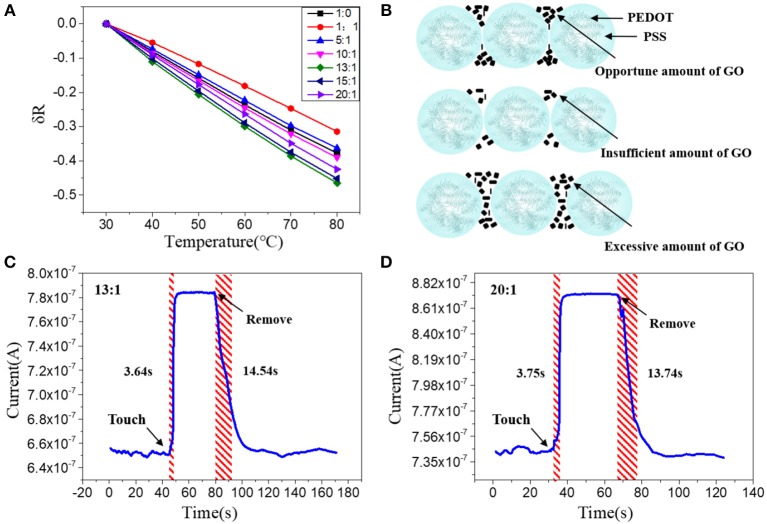
**(A)** Relative resistance changes of the sensors with different mixing ratios at different temperatures. **(B)** Schematic diagram of PEDOT: PSS nanowires doped with opportune, insufficient and excessive amount of GO; Real-time response of the nanowire sensors with a mixing ratio of **(C)** 13:1 and **(D)** 20:1, after attached to and removal off the human arm.

As discussed above, nanoscale confinement strategy can enhance the response speed of the PEDOT: PSS to temperature and PEDOT: PSS nanowires with the highest S/V ratio show the fattest response to temperature change which is rather important to develop as temperature sensor. Next, we focus on improving its thermal sensitivity. As reported before, doping of PEDOT: PSS with other temperature-sensitive materials such as graphene (Trung et al., 2014, 2016, 2018) can further increase its response to temperature (Honda et al., 2014; Oh et al., 2018). Here, we applied the graphene doping strategy to further adjust the temperature sensitivity of PEDOT: PSS nanowires. Since the mixtures are required to be dispersed in aqueous solution for capillary filling, the oxidation form of graphene (GO) was used, which contains many oxygen-containing functional groups, such as hydroxyl, epoxide and carboxyl groups and ensures its good solubility in aqueous solution (Wang et al., 2009; Chen and Li, 2012; Lee et al., 2012; Dwandaru et al., 2019). The doped mixtures were characterized by FT-IR (Figure S2 in Supporting Information), the mixtures have the same characteristics as PEDOT: PSS and contain the specific bands of GO, which proves that the two materials have been fully mixed and the chemical structures of PEDOT: PSS and GO are well-maintained. We then optimized the GO doping ratio to understand its thermal sensing effect. I–V curves of the nanowires constructed by mixtures with different mixing ratios (PEDOT: PSS/GO, V/V) were measured under temperature range from 30 to 80°C with an interval of 10°C. As shown in Figure 3A, the GO doping has a conspicuous influence on the thermal sensitivity of the PEDOT: PSS. The nanowire device with mixing ratio of 13:1 shows the highest temperature response. This can be explained by the fact that GO is a kind of relatively hydrophilic material, thus GO should be mostly adsorbed on the hydrophilic PSS when it is mixed with PEDOT: PSS. Hence a little bit of GO will not affect the overall conductivity of PEDOT: PSS due to PSS does not participate in conductivity of PEDOT: PSS. However, GO has excellent thermal conductivity (Teng et al., 2011; Yao et al., 2016), hence doping GO can affect the thermal sensitivity of PEDOT: PSS. When the mixing ratio is 13:1 (The upper schematic of Figure 3B), the GO flakes are fully filled in the PSS and the gap between the adjacent PEDOT: PSS nanoparticles. Such composite materials show higher temperature sensitivity compared to the less, more or no GO filling. If the mixing ratio is higher than 13:1, such as 15:1, which means the amount of GO flakes is less than that of 13:1, hence the GO flakes are not adequately filled in the PSS and the gap between adjacent PEDOT: PSS nanoparticles(The middle schematic of Figure 3B), leading to the temperature sensitivity of this ratio lower than that of 13:1 but higher than non-doped pure PEDOT: PSS. If the mixing ratio is lower than 13:1, such as 10:1, under such mixing ratio, the amount of GO is larger than that of 13:1, hence the connection between adjacent PEDOT: PSS nanoparticles will be affected (The bottom schematic of Figure 3B), leading to the response of this ratio lower than that of 13:1. Furthermore, when the ratio is further reduced, such as 1:1, which means the proportion of GO is far more than that of 13:1, hence the connection between the PEDOT: PSS nanoparticles will be affected severely, leading to the response of this ratio is even lower than that of non-doped pure PEDOT: PSS. We also explored whether the GO doping would affect the response speed of PEDOT: PSS nanowires. As shown in Figures 3C,D, compared with the pure PEDOT: PSS nanowires (Figure 2E), the response time and recovery time of these GO doped nanowires have no obvious difference. It proves that the GO doping did not affect the response speed of PEDOT: PSS nanowires but only enhance the thermal sensitivity. Thus, by combining the nanoconfinement and GO doping strategy, the response speed and thermal sensitivity can be simultaneously optimized. The 13:1 GO doped nanowires are used to prepare the temperature sensor for rest of the applications.

### Repeatability and Stability

**Figure 4:**
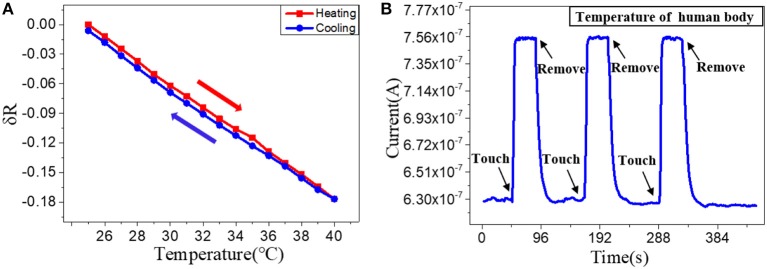
**(A)** Relative resistance changes depending on the temperature with increments/decrements of 1°C. **(B)** Real-time response of a nanowire sensor after attached to and removal off the human arm for three times.

After optimization the structure and the materials, we then fabricated the optimized Go doped PEDOT: PSS nanowires on a flexible PET substrate to facilitate their epidermal sensing applications. The repeatability and stability of the nanoscale flexible sensors were tested, especially for their hysteretic behavior (Han et al., 2018). Figure 4A shows the hysteresis of the nanowires response to temperature with a heating and cooling cycle, which indicates a very minor hysteresis (~0.0046). Next, we evaluated the repeatability of the nanowires-based sensor by applying the sensor for multiple tests of touch to and removal from the skin (Figure 4B). The stable performance demonstrates that the devices have good repeatability and stability. The sensors were then kept under ambient air for 1 months and their response to temperature change were measured again. The results in Figure S3 in the Supporting Information show that their temperature response after 1 months is slightly changed in comparison with the as-fabricated sensors, which prove the long-term stability of the PEDOT: PSS nanowires in ambient air.

### Skin Temperature Detection

**Figure 5:**
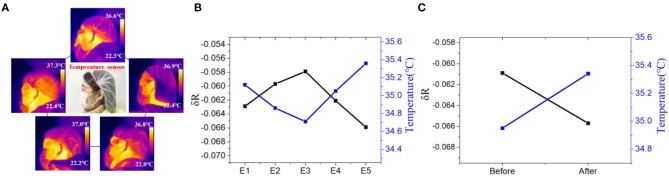
**(A)** IR thermograms and picture(middle) of the temperature sensor attached on the head of five volunteers. Resistance of the device and measured skin temperature of **(B)** five volunteers and **(C)** a volunteer before and after 30 push-ups.

We then tested the nanowires-based temperature sensor for skin temperature detection. Five volunteers were chosen to use the sensors for monitoring their local skin temperature by attaching these devices on their heads (Figure 5A). The good mechanical property and ultra-thin size of the flexible sensor facilitate its attachment to human skin and hence ensure the reliable temperature measurement. According to the results of the previous fitting, the corresponding skin temperature of five volunteers can be calculated. As shown in Figure 5B, the skin temperature of the five volunteers is 35.12°C, 34.86°C, 34.71°C, 35.05°C, 35.36°C, respectively. The results are consistent with the results obtained by thermocouple which proves the accuracy of the device (35.1°C, 34.7°C, 34.7°C, 35.0°C, 35.2°C). Since the skin temperature will change during strenuous or long-term exercise, we apply the temperature sensor to track the volunteer exercise status. The sensor was attached to the head of a volunteer to monitor the subtle temperature changes during exercise. The normal skin temperature of the volunteer's temple was measured to be approximately 34.93°C. It increased to approximately 35.37°C after doing 30 push-ups (see Figure 5C). These results demonstrate that the nanowire-based temperature sensors can be effectively applied for rapidly and accurately tracking of human skin temperature.

### Mobile Healthcare Based on Nanoscale Fees

**Figure 6:**
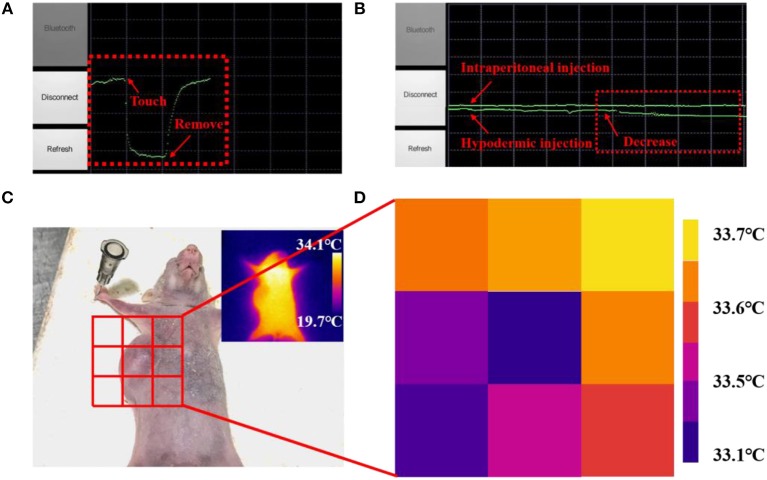
A screenshot of the smartphone when the sensor was attached on **(A)** a human arm, **(B)** a mouse after intraperitoneal injection of adrenaline and a mouse after hypodermic injection of adrenaline. **(C)** Picture of the mouse with a subcutaneous tumor. **(D)** Skin temperature distribution of subcutaneous tumor and eight points near it measured by the nanowires-based EES.

Many diseases and physiological behaviors will cause local changes in body epidermal temperature, hence real-time and continuous measurement of the local skin temperature could enable a better tracking of personal health status (Deng and Liu, 2004; Helmy and Rizkalla, 2008; Ng, 2009; Li et al., 2017). Based on the nanoscale flexible temperature sensor, a wearable FEES was further developed by integrating the nanowires with commercial electronic components to enable the wireless communication. An Android application program was further developed and installed on a smartphone to directly receive and process the sensing signals in real-time. As shown in Figure S4, the FEES can be worn on the wrist for continuously monitoring the skin temperature. Figure 6A shows the screenshot of the smartphone interacting with the FEES system by touching or removing the system from the skin, which demonstrates their rapid response to human skin temperature. The system was further applied to compare the administration route of drugs by monitoring the subtle skin temperature changes after intraperitoneal and hypodermic injection of adrenaline to a mouse (Figure 6B). It clearly shows that after intraperitoneal injection of adrenaline, the resistance of the device did not change significantly, indicating that body temperature of the mouse was constant. While after hypodermic injection, the resistance of the sensor was slowly and continuously decreased, indicating that body temperature of the mouse was slowly increasing, which are consistent with the existing studies (Maling et al., 1979). These results demonstrate the feasibility of the FEES system in real temperature monitoring applications.

Subcutaneous tumors, as a kind of common tumor, will change the metabolic activity of the lesion area and in turn change the normal temperature distribution on the skin surface (Sudharsan et al., 1999). Benefited by the fast response and high thermal sensitivity, the EES could provide a neat tool to monitoring skin temperature outside the subcutaneous tumors in a new way. Figure 6D shows the skin temperature distribution around the tumor area detected by attaching the FEES on different place of the skin (Figure 6C). The results indicate that the temperature in the tumor area is significantly lower than the surrounding area, which is consistent with the results of existing studies (Konerding and Steinberg, 1988). Meanwhile, the results detected by the FEES are more accurate than IR thermograms (inset in Figure 6C), and this method provides the possibility of real-time monitoring. It also shows that the sensor has great potential to be made into temperature sensor arrays, which can detect spatial mapping of skin temperature so that the arrays could provide a feasible method to judge the diffusion area and monitor the deterioration status of the subcutaneous tumors efficiently and conveniently. All these results demonstrate that this EES has great potential as a wearable bioelectronic for mobile medical diagnosis and healthcare applications.

## Conclusion

In this work, we developed a strategy by combining the micro/nano confinement with materials doping to enable simultaneous optimization of the response speed and sensitivity of low dimension conductive polymers. By confining the PEDOT: PSS into nanowires and doping with GO at optimized doping ratio, ultra-fast response to temperature change (<3.5 s) and maximized thermal-sensitivity is achieved. Well-defined sub-100 nm nanowires were fabricated on flexible substrates using a low-cost nanoscale printing approach which were further integrated as a functional skin-attachable flexible epidermal electronic system (FEES) to enable a live and wireless temperature sensing. The developed FEES were applied for different physiological behaviors and diseases monitoring by recording the real-time skin temperature changes.

## Data Availability Statement

All datasets generated for this study are included in the article/Supplementary Material.

## Ethics Statement

The animal study was reviewed and approved by the ethics committee of the Institute of Radiation Medicine Chinese Academy of Medical Sciences.

## Author Contributions

CZ developed the ideas of the study, carried out most of the experiments, and also wrote most parts of the manuscript. NT and XZ also developed some ideas of the study and participated in some experiments, and also reviewed the manuscript. YF, YJ, and HZ participated in some experiments of the study and reviewed the manuscript. XD developed some ideas and improved the ideas other authors developed and also wrote parts of the manuscript. All authors read and approved the final manuscript.

### Conflict of Interest

The authors declare that the research was conducted in the absence of any commercial or financial relationships that could be construed as a potential conflict of interest.
