# Supplementary material for: Simultaneously Optimize the Response Speed and Sensitivity of Low Dimension Conductive Polymers for Epidermal Temperature Sensing Applications
Source: Front Chem. 2020 Mar 19;8:194. doi: 10.3389/fchem.2020.00194 (PMC7098694; doi:10.3389/fchem.2020.00194)
Supplement: Supplementary file 1 [file Data_Sheet_1.pdf]

Supporting Information

Simultaneously Optimize the Response Speed and Sensitivity of Low Dimension Conductive Polymers for Epidermal Temperature Sensing Applications

Cheng Zhou, Ning Tang, Xiaoshuang Zhang, Ye Fang, Yang Jiang, Hainan Zhang, Xuexin Duan *

State Key Laboratory of Precision Measuring Technology & Instruments, Tianjin University, Tianjin 300072, China

*Corresponding author. Tel. /Fax: +86 2227401002. E-mail: xduan@tju.edu.cn

Supporting Figures.

Figure S1. Response of PEDOT: PSS film to temperature.

Figure S2. FT-IR Spectra.

Figure S3. Long-term Stability.

Figure S4. Homemade wearable system.

1. The comparison between our method and current available methods for fabricating nanowires

Methods	Process	Cost	Orientation	Minimum linewidth
Physical vapor deposition [1-2]	Time-consuming	High	Irregular	Over-100nm
Self-assembly [3-4]	Time-saving	Low	Regular	Over-100nm
Ink-jet printing [5-6]	Time-saving	High	Regular	Over-100nm
Ion beam lithography[7-8]	Time-saving	High	Regular	Sub-100nm
Electrospinning method [9-10]	Time-saving	High	Irregular	Sub-100nm
Stretch spinning method [11]	Time-saving	Low	Irregular	Sub-100nm
Electrodeposition [12]	Time-saving	High	Regular	Sub-100nm
Atomic force microscope scratch [13]	Time-consuming	High	Regular	Sub-100nm
**Soft nanolithography (this work)**	**Time-saving**	**Low**	**Regular**	**Sub-100 nm**

2. Response of PEDOT: PSS film to temperature

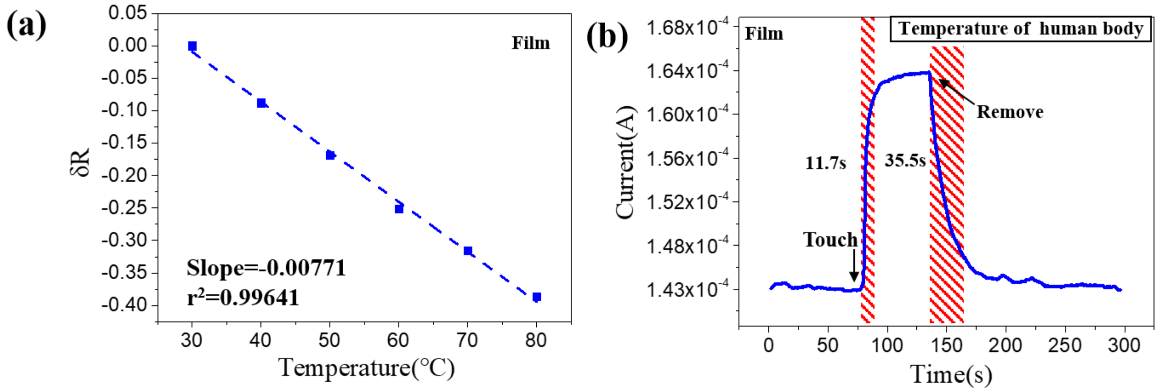

Figure S1. (a) Relative resistance changes depending on the temperature with increments of 10°C of the PEDOT:PSS film based temperature sensor; (b) Real-time response of PEDOT: PSS film based temperature sensor.

3. FT-IR Spectra

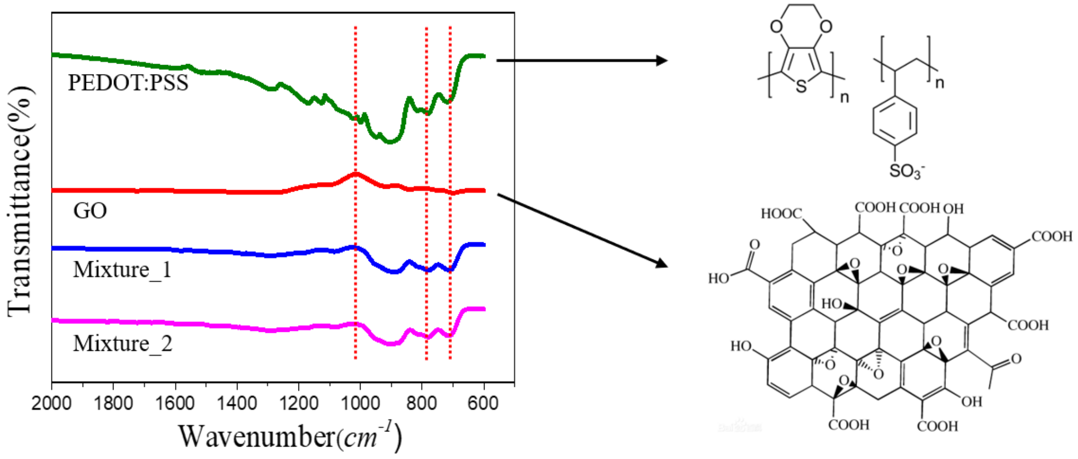

Figure S2. FT-IR spectra of PEDOT:PSS, GO and products obtained after mixing with different weight percentages of GO. 25% (Mixture1), 50%(Mixture2), (GO/PEDOT: PSS, V/V).

4. Long-term Stability

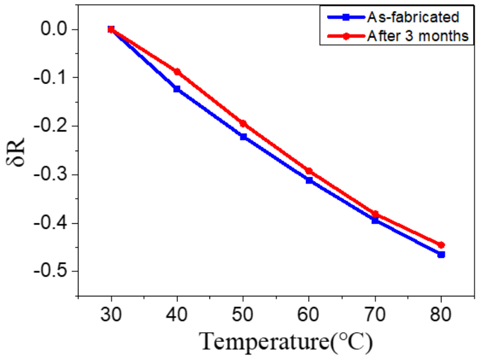

Figure S3. The responsivity of as-fabricated nanowires-based sensor and after being kept in ambient air for 1 months to temperature.

5. Homemade wearable system

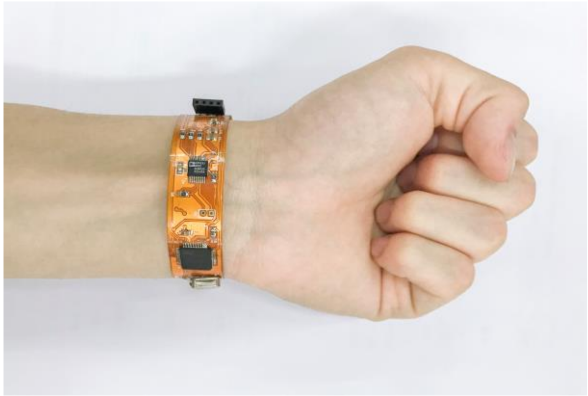

Figure S4. A photograph of the homemade wearable system worn on a wrist.

References

[1] Singh J, Wolfe D E. Review Nano and macro-structured component fabrication by electron beam-physical vapor deposition (EB-PVD) [J]. Journal of materials Science, 2005, 40(1): 1-26.

[2] Zhou X, Gan L, Zhang Q, et al. High performance near-infrared photodetectors based on ultrathin SnS nanobelts grown via physical vapor deposition[J]. Journal of Materials

Chemistry C, 2016, 4(11):2111-2116

[3] Giri G, Park S, Vosgueritchian M, et al. High-mobility, aligned crystalline domains of TIPS-pentacene with metastable polymorphs through lateral confinement of crystal growth[J]. Advanced Materials, 2014, 26(3): 487-493.

[4] Zhang C, Zhang X, Zhang X, et al. Facile One-Step Growth and Patterning of Aligned Squaraine Nanowires via Evaporation-Induced Self-Assembly[J]. Advanced Materials, 2008, 20(9): 1716-1720

[5] Noh Y Y, Cheng X, Sirringhaus H, et al. (2007). Ink-jet printed ZnO nanowire field effect transistors[J]. Applied Physics Letters, 2007,91(4): 043109.

[6] Cui Z, Han Y, Huang Q, et al. Electrohydrodynamic printing of silver nanowires for flexible and stretchable electronics[J]. Nanoscale, 2018,10(15): 6806-6811.

[7] Bhuvana T, Kulkarni G U. Highly conducting patterned Pd nanowires by direct-write electron beam lithography[J]. ACS nano, 2008, 2(3): 457-462.

[8] Llobet J, Rius G, Chuquitarqui A, et al. Arrays of suspended silicon nanowires defined by ion beam implantation: mechanical coupling and combination with CMOS technology[J]. Nanotechnology, 2018,29(15):155303.

[9] Hosono E, Wang Y, Kida N, et al. Synthesis of triaxial LiFePO4 nanowire with a VGCF core column and a carbon shell through the electrospinning method[J]. ACS applied materials & interfaces, 2009,2(1): 212-218.

[10] Zhu C, Yu Y, Gu L, et al. Electrospinning of Highly Electroactive Carbon-Coated

Single - Crystalline LiFePO4 Nanowires[J]. Angewandte Chemie International Edition, 2011,50(28): 6278-6282.

[11] Bai X, Liao S, Huang Y, et al. Continuous draw spinning of extra-long silver submicron fibers with micrometer patterning capability[J]. Nano letters, 2017,17(3): 1883-1891.

[12] Yin A J, Li J, Jian W, et al. Fabrication of highly ordered metallic nanowire arrays by electrodeposition[J]. Applied Physics Letters, 2001,79(7): 1039-1041.

[13] Lu H, Lin, Hsiao, T, , et al. Electrical properties of single and multiple poly (3, 4-ethylenedioxythiophene) nanowires for sensing nitric oxide gas[J]. Analytica chimica acta, 2009, 640(1-2): 68-74.
